# Long-term outcome of patients undergoing pacemaker implantation after transcatheter aortic valve implantation: a systematic review and meta-analysis

**DOI:** 10.1007/s12928-025-01232-4

**Published:** 2026-01-09

**Authors:** Cecilia Veraar, Gudrun Lamm, Lion Merl, Arabella Fischer-Hammerschmied, Matthias Granner, Maximilian Will, Konstantin Schwarz, Andreas Kammerlander, Julia Mascherbauer

**Affiliations:** 1 Karl Landsteiner University of Health Sciences, Dr. Karl-Dorrek-Straße 30, Krems, 3500 Austria; 2 Division of Internal Medicine 3, University Hospital St. Pölten, Dunant- Platz 1, St. Pölten, 3100 Austria; 3 Department of Anesthesiology, Intensive Care Medicine and Pain Medicine, Division of Cardiac Thoracic Vascular Anesthesia and Intensive Care Medicine, Medical University of Vienna, Vienna, Austria; 4 Department of Cardiology, Medical University of Vienna, Vienna, Austria

**Keywords:** Transcatheter aortic valve implantation, Permanent pacemaker, Long-Term mortality, Meta-Analysis, Prognosis

## Abstract

The long-term prognostic relevance of permanent pacemaker (PM) implantation after transcatheter aortic valve implantation (TAVI) remains uncertain. We performed a meta-analysis to evaluate its association with all-cause mortality beyond five years. Following MOOSE recommendations, PubMed and Embase were searched through September 2025 for studies reporting long-term outcomes after TAVI, including hazard ratios (HRs) comparing patients with and without new PM implantation. Pooled HRs were calculated using random-effects models with restricted maximum likelihood estimation and Hartung–Knapp adjustment. Heterogeneity was quantified using I², and robustness was tested by leave-one-out procedures and alternative model estimators. Random-effects meta-regression examined whether study-level covariates modified the association. Seven observational studies comprising 59 635 patients were included, of whom 11 325 (19%) received a new PM within 30 days after TAVI. Follow-up ranged from 60 to 120 months. PM implantation was associated with higher long-term all-cause mortality {pooled HR 1.13 (95% CI 1.07–1.19); I² = 0%}. Leave-one-out analyses confirmed stability (HR 1.11–1.13), and funnel-plot inspection revealed no asymmetry. None of the examined covariates—female sex, diabetes, atrial fibrillation, conduction disturbances, coronary artery disease, or left-ventricular ejection fraction—significantly affected the association (β range − 0.018 to + 0.024; exp(β) 0.98–1.02; all *p* > 0.10). Across seven contemporary registries, PM implantation after TAVI was consistently linked to a modest increase in long-term mortality, independent of baseline comorbidities, emphasizing the need for conduction-preserving implantation and physiologic pacing strategies.

**Graphical abstract:**

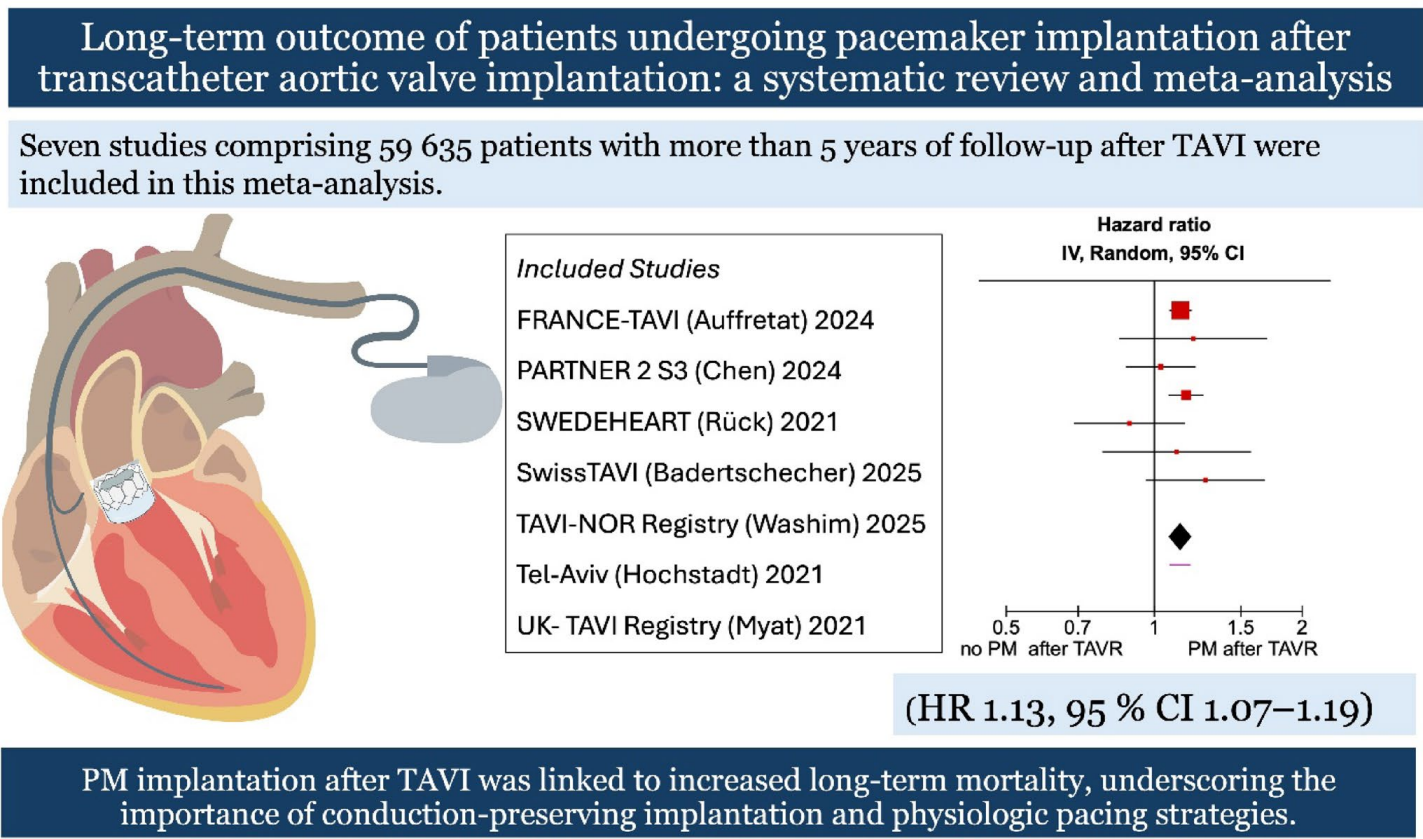

**Supplementary Information:**

The online version contains supplementary material available at 10.1007/s12928-025-01232-4.

## Introduction

Transcatheter aortic valve implantation (TAVI) has rapidly evolved over the past two decades from a treatment option for inoperable patients to the preferred therapy across most surgical risk profiles [1–3]. Despite substantial progress in valve technology and implantation techniques, conduction disturbances remain frequent post-procedural complications, often necessitating permanent pacemaker (PM) implantation in 10–30% of cases [4, 5]. 

The clinical relevance of PM after TAVI has been extensively investigated for short-term outcomes, with several meta-analyses evaluating its impact on 1-year mortality, yet results remain conflicting [6, 7]. 

Most prior meta-analyses were restricted by limited follow-up durations, heterogeneous definitions of clinical endpoints, and variable adjustment for confounders [8]. With the progressive extension of TAVI to younger and lower-risk patients, attention is shifting toward long-term durability and late outcomes. In this context, clarifying whether PM implantation influences survival years after the procedure is of growing importance [9]. 

Crucially, no meta-analysis has yet addressed long-term outcomes ≥ 5 years, leaving the prognostic significance of PM after TAVI over extended follow-up uncertain.

We therefore conducted a systematic review and meta-analysis to assess the association between PM after TAVI and all-cause mortality at five years and beyond, synthesizing evidence from large-scale observational studies and contemporary registries.

## Methods

This meta-analysis was conducted in accordance with the Meta-analysis Of Observational Studies in Epidemiology (MOOSE) guidelines and was prospectively registered in the PROSPERO International Prospective Register of Systematic Reviews (ID 1158286) [10]. 

### Search strategy and selection criteria

A systematic and comprehensive literature search was performed in PubMed and Embase through September 2025 to identify studies evaluating the association between PM implantation after TAVI and long-term all-cause mortality ≥ 5 years. A combination of keywords and MeSH terms, including TAVI, PM, and mortality, was used. The complete search strategy is provided in Supplementary Table S1.

All records were independently screened by CV and LM at the title and abstract level, with discrepancies resolved by discussion and consensus.

Studies were eligible for inclusion if they met the following criteria:

Reported on clinical outcomes after PM implantation following TAVI in native aortic valves, provided all-cause mortality data at ≥ 5 years of follow-up and reported hazard ratios (HRs) with 95% confidence intervals for all-cause mortality.

In all included registries and cohorts, patients with pre-existing PM were excluded from the comparative analysis. Consequently, Table 1 displays only those without prior PM, subdivided into new PM (within 30 days) and no PM. By contrast, Table 3 reports the total procedural numbers per study, including patients with pre-existing PM, to provide full registry-level valve distributions. The primary endpoint of this study was all-cause mortality at ≥ 5 years after TAVI.

**Table 1:** Baseline characteristics of included studies. Characteristics of included studies reporting ≥ 5-year all-cause mortality after TAVI with and without PM

Author (Year)	Type of Study	Region	Centers	Inclusion Period	Number of Patients	New PM (%)	Timing of PM	Follow-up (months)
Auffret (2024) FRANCE-TAVI	Observational	France	48 centers (multicenter)	2013–2019	34,717	6,973 (20.1)	≤ 30 days	60
Chen (2024) PARTNER 2 S3	Observational	USA	51 centers (multicenter)	2014–2017	857	107 (12.5)	≤ 30 days	60
Rück (2021) SWEDEHEART	Observational	Sweden	7 centers (nationwide)	2008–2018	3,420	481 (14.1)	≤ 30 days	120
Badertscher (2025) SwissTAVI	Observational	Switzerland	11 centers (multicenter)	2011–2022	11,994	2,028 (16.9)	≤ 30 days	120
Wasim (2025) TAVI-NOR	Observational	Norway	Single- center	2012–2019	548	173 (31.6)	≤ 30 days	84
Hochstadt (2021) Tel-Aviv	Observational	Israel	Single-center	2009–2019	1,284	254 (19.8)	≤ 30 days	72
Myat (2021) UK-TAVI	Observational	UK	93 centers (nationwide)	2007–2015	6,815	1309 (19.2)	≤ 30 days	84

All patients with pre-existing PM implantation prior to TAVI were excluded from the comparative analysis. Therefore, this table includes only patients without prior PM, subdivided into those who received a new PM within 30 days and those without PM

PM, Pacemaker; TAVI, transcatheter aortic valve implantation; HR, Hazard Ratio

### Data extraction and quality assessment

Two authors (CV, LM) independently extracted study characteristics, including author, year, design, sample size, timing and incidence of PM implantation, baseline demographics, valve type, follow-up duration, and HRs with 95% CIs for ≥ 5-year all-cause mortality. Study quality was assessed using the Newcastle–Ottawa Scale (NOS) (0–9 points).

### Statistical analysis

Effect estimates were extracted as hazard ratios (HRs) with corresponding 95% confidence intervals (CIs). HRs were transformed into log(HR) values, and standard errors were derived from the reported CIs. Pooled effect estimates were calculated using random-effects models with the restricted maximum likelihood (REML) method. To provide more conservative inference, confidence intervals and p-values were adjusted using the Hartung–Knapp approach.

The Hartung–Knapp adjustment was applied, due to the small number of included studies. In addition, a 95% prediction interval was calculated to quantify the expected range of true effects in comparable studies.

Between-study heterogeneity was quantified using the I² statistic and τ², and a 95% prediction interval was reported. Sensitivity analyses included leave-one-out procedures and model comparisons (DerSimonian–Laird estimator and fixed-effect model). Meta-regression under a random-effects framework was performed to explore potential effect modification by study-level covariates, including age, female sex, diabetes mellitus (DM), atrial fibrillation (AF), right and left bundle-branch block (RBBB, LBBB), and coronary artery disease (CAD). All analyses were performed in R (version 2025.05.1 + 513; R Foundation for Statistical Computing, Vienna, Austria).

## Results

### Study selection

**Fig. 1:**
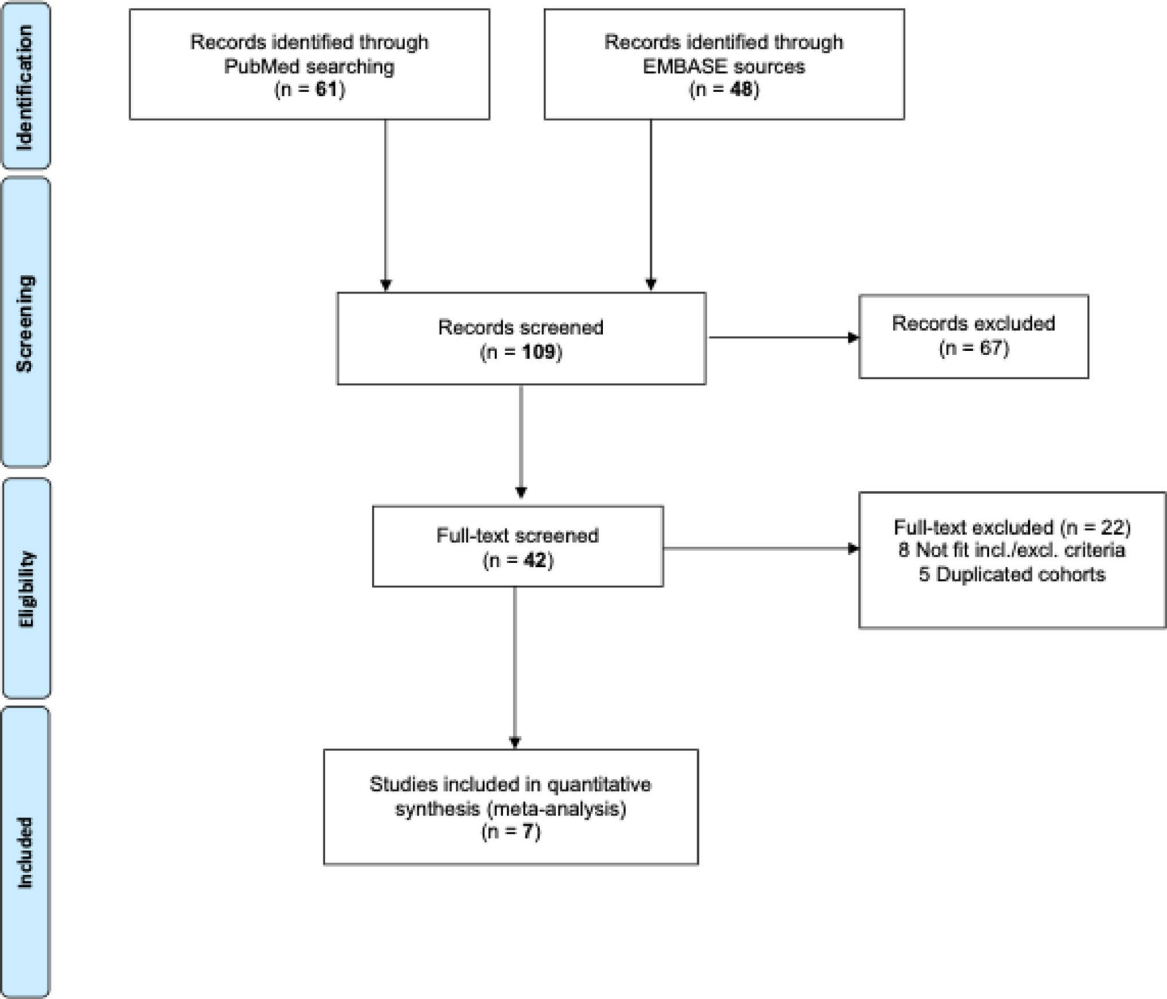
MOOSE Flow Chart of literature search. Showing the number of records identified through database searching, exclusions at different stages, and the final number of studies included in the meta-analysis

The systematic search yielded a total of 109 records (61 from PubMed and 48 from Embase). After title and abstract screening, 67 records were excluded. Of the remaining 42 full-text articles assessed for eligibility, 35 full-texts were excluded: 22 after detailed review, 8 that did not meet the inclusion/exclusion criteria, and 5 with duplicated cohorts. Finally, 7 published studies were included in the analysis as depicted in Fig. 1.

### Study characteristics

Seven included observational studies comprised a total of 59,635 patients undergoing TAVI, with enrollment periods ranging from 2008 to 2022. The cohorts represented both single-center experiences (Norway, Israel) and large multicenter or nationwide registries (France, Switzerland, Sweden, United Kingdom, USA). Sample sizes varied considerably, ranging from 548 patients in the Norwegian single-center study to 34,717 participants in the FRANCE-TAVI registry. The incidence of PM implantation within 30 days after TAVI ranged from 12.5% to 31.6% across studies. Corresponding to an overall pooled incidence of 19%. Follow-up for mortality outcomes extended between 60 and 120 months as detailed in Table 1.

### Patient characteristics

Patient characteristics of seven large national and multicenter TAVI cohorts (FRANCE-TAVI, PARTNER 2 S3, SWEDEHEART, SwissTAVI, TAVI-NOR, Tel-Aviv, UK-TAVI) were shown in Table 2 [11–14]. The incidence of new PM implantation ranged from 12.5% in PARTNER 2 S3 to 31.6% in TAVI-NOR. Among patients with PM, the proportion of women varied between 41.3% in UK-TAVI and 63.6% in PARTNER 2 S3, compared with 47.3% in UK-TAVI and 59.0% in Tel-Aviv among those without PM. DM prevalence ranged from 2.1% in SwissTAVI to 39.7% in Tel-Aviv in the PM group, and from 2.3% in SwissTAVI to 34.3% in Tel-Aviv in the non-PM group. CAD, reported in selected studies, ranged from 14.4% in SwissTAVI to 82.2% in PARTNER 2 S3 among PM patients, and from 15.3% in SwissTAVI to 74.8% in PARTNER 2 S3 among non-PM patients. AF was observed in 11.9% of PM patients in SwissTAVI and 43.7% in SWEDEHEART, compared with 12.3% and 39.6% in the corresponding non-PM groups. Left ventricular ejection fraction was broadly preserved across studies in both groups (55–59%). Baseline conduction disturbances were less consistently reported: RBBB ranged from 2.3% in UK-TAVI to 7% in TAVI-NOR among non-PM patients, whereas LBBB ranged from 1.9% in PARTNER 2 S3 to 12% in TAVI-NOR in the non-PM group, and from 2.2% in PARTNER 2 S3 to 8% in TAVI-NOR in the PM group.

**Table 2:** Baseline data of patients included in each study. Demographic and clinical characteristics of patients included in each study, stratified by PM status

Variable		FRANCE-TAVI	PARTNER 2 S3		SWEDE- HEART	SwissTAVI	TAVI-NOR	Tel-Aviv	UK-TAVI
n	No PM	27,744 (79.9)	750 (87.5)	2,939 (85.9)		9,966 (83.1)	375 (68.4)	1,030 (80.2)	5,506 (80.7)
	PM	6,973 (20.1)	107 (12.5)	481 (14.1)		2,028 (16.9)	173 (31.6)	254 (19.8)	1,309 (19.2)
Female	No PM	14,801 (53.3)	317 (57.7)	1,511 (51.4)		4,971 (49.8)	195 (52.0)	608 (59.0)	2,606 (47.3)
	PM	3,134 (44.6)	39 (63.6)	211 (43.9)		850 (41.9)	82 (47.0)	133 (52.4)	540 (41.3)
DM	No PM	7,050 (25.4)	254 (33.9)	830 (28.2)		237 (2.3)	34 (20.0)	349 (34.3)	1,268 (23.0)
	PM	1,996 (28.6)	32 (29.9)	158 (32.8)		44 (2.1)	65 (17.0)	100 (39.7)	315 (24.0)
CAD	No PM	N.A	561 (74.8)	N.A		1,517 (15.2)	262 (70.0)	518 (51.0)	N.A
	PM	N.A	88 (82.2)	N.A		292 (14.4)	126 (73.0)	138 (54.8)	N.A
AF	No PM	7,504 (29.5)	263 (35.1)	1,165 (39.6)		1,233 (12.3)	96 (26.0)	252 (24.8)	1,290 (23.4)
	PM	2,157 (33.3)	36 (33.6)	210 (43.7)		243 (11.9)	67 (39.0)	80 (31.7)	297 (22.7)
EF	No PM	57.0 ± 12.8	59.9 ± 12.4	N.A		56.6 ± 12.7	57.0 ± 10.6	57.0 ± 6.4	N.A
	PM	56.9 ± 12.7	58.7 ± 12.9	N.A		55.8 ± 12.8	56.9 ± 9.3	56.9 ± 6.8	N.A
RBBB	No PM	N.A	41 (6.1)	N.A		N.A	25 (7.0)	47 (5.7)	129 (2.3)
	PM	N.A	28 (30.4)	N.A		N.A	21 (12.0)	33 (16.4)	101 (7.7)
LBBB	No PM	N.A	13 (1.9)	N.A		N.A	20 (12.0)	74 (8.9)	251 (4.6)
	PM	N.A	2 (2.2)	N.A		N.A	30 (8.0)	14 (7.0)	60 (4.6)

AF, atrial fibrillation; AUT, Austria; CAD, coronary artery disease; EF, ejection fraction; DM, Diabetes Mellitus; LBBB, left bundle branch block; N.A., not available; PM, pacemaker; RBBB, right bundle branch block; TAVI, transcatheter aortic valve implantation; UK, United Kingdom

### Heart valve types

Valve type distribution is summarized in Table 3. Marked differences were observed across countries. Balloon-expandable valves predominated in FRANCE-TAVI (62%) and UK-TAVI (55%), whereas self-expanding valves were dominant in Tel-Aviv (62%), SWEDEHEART, and TAVI-NOR (70%). SwissTAVI showed a nearly balanced use (49%) balloon-expandable vs. 48% self-expanding), while mechanically expanding valves were uncommon, reaching 4% in UK-TAVI, 2.5% in SwissTAVI, and 18% in TAVI-NOR. PARTNER 2 S3 exclusively reported balloon-expandable valves (100%).

**Table 3:** Valve type distribution stratified by PM status across included studies. Distribution of valve prostheses across studies stratified by PM status. Balloon-expandable valves

	*n*	Balloon-expandable	Self-expanding	Mechanically-expanding
SwissTAVI	Total	**6**,**617 (49.6)**	**6**,**386 (47.9)**	**338 (2.5)**
	No PM	5,151 (51.8)	4,617 (46.4)	182 (1.8)
	PM	849 (41.9)	1,059 (52.3)	118 (5.8)
France-TAVI	Total	**22**,**570 (61.8)**	**13**,**893 (38.1)**	N.A
	No PM	19,060 (64.5)	10,432 (35.3)	N.A
	PM	3,510 (50.2)	3,461 (49.4)	N.A
SWEDEHEART	Total	**1**,**309 (38.4)**	N.A	N.A
	No PM	1,212 (41.3)	N.A	N.A
	PM	97 (20.2)	N.A	N.A
UK-TAVI	Total	**3**,**732 (55.2)**	**2**,**748 (40.6)**	**273 (4.0)**
	No PM	3,270 (59.4)	2,025 (36.8)	160 (2.9)
	PM	460 (35.1)	723 (55.2)	113 (8.6)
Tel-Aviv	Total	**N.A**	**799 (62.2)**	**N.A**
	No PM	N.A	633 (61.8)	N.A
	PM	N.A	166 (65.4)	N.A
Partner 2 S3	Total	**857 (100)**	-	-
	No PM	750 (100)	-	-
	PM	107 (100)	-	-
TAVI-NOR	Total	**65 (11.8)**	**385 (70.2)**	**98 (17.9)**
	No PM	63 (16.8)	272 (72.5)	40 (10.7)
	PM	2 (1.2)	113 (65.3)	58 (33.5)

Totals include patients with pre-existing PM. PM, pacemaker

### All-cause death at long-term follow-up

**Fig. 2:**
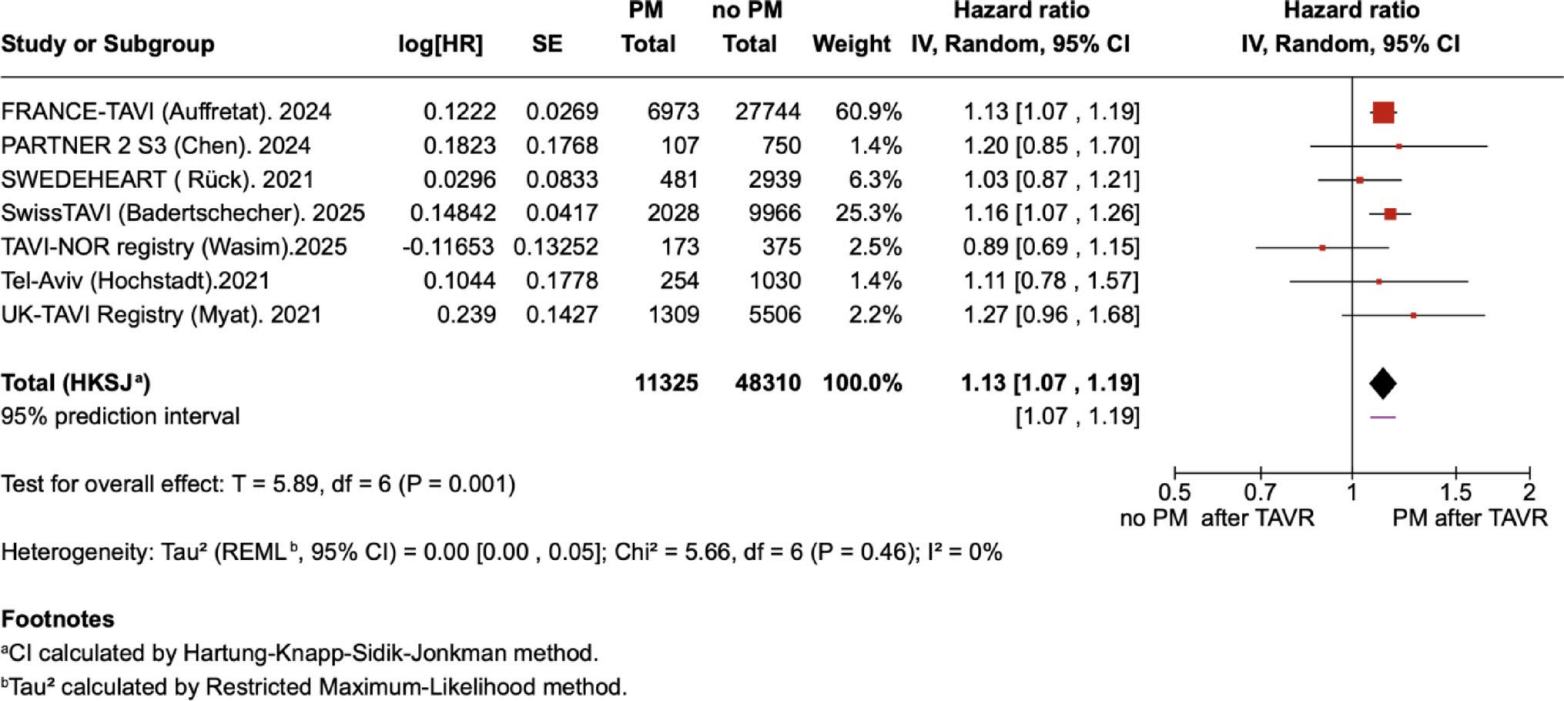
Forest plot of all-cause mortality at ≥ 5 years in patients with versus without PM after TAVI. The plot presents individual study estimates and the pooled HR with 95% CI intervals. Heterogeneity was low (I² = 0%) as indicated. CI, Confidence Interval; HR, Hazard Ratio; PM, Pacemaker; REML, Restricted Maximum Likelihood; SE, Standard Error

**Fig. 3:**
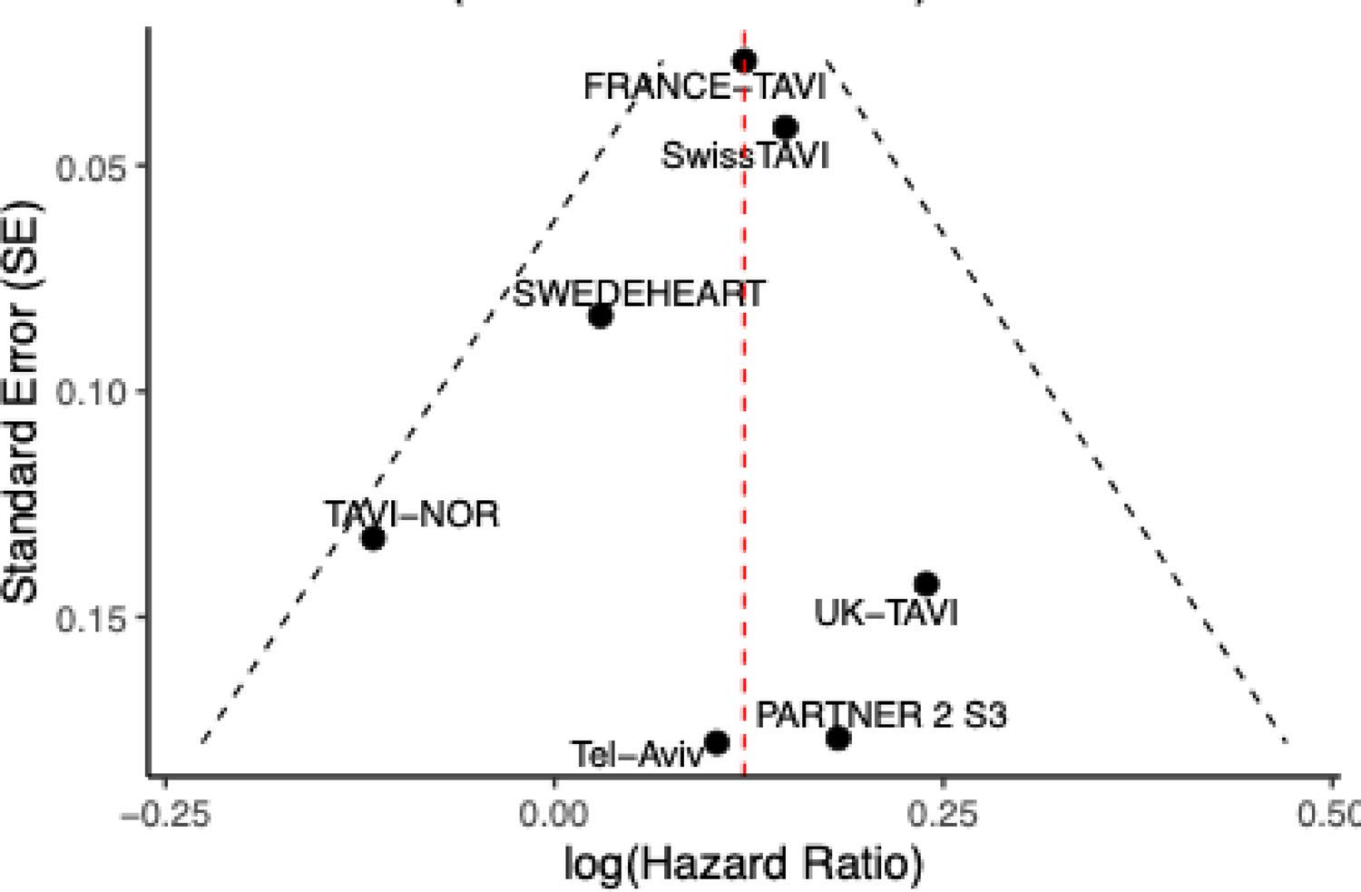
Funnel Plot of all included studies No Egger/Begg test was performed according to a limited number of included studies. Studies are plotted by log HR against precision (SE). The red dashed line shows the pooled effect estimate; black dashed curves indicate 95% limits. SE, Standard Error

Among the seven included observational studies, the risk of all-cause death at long-term follow-up was higher in patients who required permanent PM implantation compared with those who did not {HR 1.13 (95% CI 1.07–1.19); Fig. 2}. Leave-one-out sensitivity analyses showed that sequential exclusion of individual studies did not materially alter the overall effect estimate, with pooled HRs consistently ranging from 1.11 to 1.13. Heterogeneity remained very low throughout (I² = 0–17.5%), indicating that the association between PM implantation and long-term all-cause mortality was robust across studies (Table 4). Visual assessment of the funnel plot did not reveal asymmetry as depicted in Fig. 3. However, no formal Egger’s or Begg’s test was performed due to the limited number of studies.

**Table 4:** Leave-one-out sensitivity analysis of long-term mortality. This table displays pooled HRs with 95% confidence intervals and I² values after sequential exclusion of each study

Study excluded	HR (95% CI)	I² (%)
FRANCE-TAVI	1.11 (1.00–1.24)	17.5
PARTNER 2 S3	1.13 (1.06–1.19)	0.1
SWEDEHEART	1.13 (1.08–1.20)	0.0
SwissTAVI	1.11 (1.03–1.20)	4.5
TAVI-NOR	1.13 (1.09–1.18)	0.0
Tel-Aviv	1.13 (1.06–1.20)	0.1
UK-TAVI	1.12 (1.07–1.19)	0.0

CI, Confidence Interval; HR, Hazard Ratio

### Meta-regression analysis

Meta-regression including female sex, diabetes, AF, RBBB, LBBB, and CAD showed no significant effect modification of the association between PM and ≥ 5-year mortality (all *p* > 0.10). Regression coefficients (β) ranged from − 0.018 to + 0.024, corresponding to exp(β) values between 0.98 and 1.02 (Table 5).

**Table 5:** Univariate meta-regression of study-level covariates and long-term mortality. Univariable meta-regression analyses evaluating the association of study-level covariates with the effect of PM implantation on long-term all-cause mortality

Covariate	β	SE	95% CI	exp(β)	*p*-value	Interpretation
Female (%)	−0.084	0.091	−0.318–0.150	0.92	0.399	per + 10%-points
DM (%)	−0.013	0.022	−0.069–0.044	0.99	0.587	per + 10%-points
AF (%)	−0.019	0.017	−0.063–0.025	0.98	0.317	per + 10%-points
RBBB (%)	−0.430	0.384	−2.080–1.220	0.65	0.380	per + 10%-points
LBBB (%)	−0.399	0.183	−1.180–0.386	0.67	0.160	per + 10%-points
CAD (%)	−0.019	0.018	−0.077–0.039	0.98	0.370	per + 10%-points
Mean LVEF (%)	0.019	0.033	−0.087–0.125	1.02	0.603	per + 1%-point

AF, atrial fibrillation; CAD, coronary artery disease; CI, 95% confidence interval; EF, ejection fraction; DM, Diabetes Mellitus; LBBB, left bundle branch block; RBBB, right bundle branch block; SE, standard error; 95%

### Risk of bias assessment

The methodological quality of the included studies was moderate to high according to the Newcastle–Ottawa Scale (NOS), with scores ranging from 7 to 9 out of a maximum of 9 points (Supplementary Table S2). FRANCE-TAVI and SwissTAVI achieved the highest ratings (9/9), driven by strong comparability, comprehensive outcome assessment, and adequate long-term follow-up. SWEDEHEART and UK-TAVI also scored highly (8/9), although both showed limitations in the adequacy of follow-up. PARTNER 2 S3, TAVI-NOR, and Tel-Aviv reached 7/9 points; in these studies, shorter or less complete follow-up and fewer comparability points were the main contributors to lower scores.

## Discussion

In this meta-analysis, PM implantation after TAVI was associated with a 13% increase in all-cause mortality at ≥ 5 years. This association was consistent across sensitivity analyses, and funnel plot inspection revealed no evidence of publication bias. Meta-regression demonstrated that none of the examined covariates significantly modified the association between PM and long-term mortality.

To our knowledge, this is the first meta-analysis focusing exclusively on long-term outcomes beyond 5 years, thereby expanding prior evidence that mainly addressed 1-year mortality. Earlier meta-analyses by Siontis et al. and Faroux et al. showed inconsistent short-term effects, whereas our findings suggest that the adverse impact of PM becomes more apparent over extended follow-up. This temporal pattern supports the notion that pacing-related ventricular dyssynchrony may exert cumulative effects on cardiac remodeling and heart failure development [4, 6, 7]. 

The included studies varied in design, population size, and device distribution. Large registries such as FRANCE-TAVI and SwissTAVI yielded precise estimates with narrow confidence intervals, strengthening the pooled analysis [11, 13]. In contrast, smaller cohorts like TAVI-NOR, characterized by a predominance of self-expanding and mechanically expanding valves, reported higher PM rates but non-significant mortality effects [14]. These discrepancies may reflect procedural and technological differences, as well as varying thresholds for PM implantation. Liberal implantation practices could dilute associations by including patients with transient conduction abnormalities.

Our study showed that considerable variability in valve selection, device generations, and national implantation practices did not result in measurable statistical heterogeneity. Across all included cohorts, the effect estimates were closely aligned, suggesting that long-term mortality differences associated with PM implantation were consistent irrespective of procedural preferences. The uniformity of these estimates likely explains the very low I² despite clear clinical variation between registries.

Chronic right ventricular pacing induces electrical and mechanical dyssynchrony, leading to adverse remodeling and progressive heart failure. These mechanisms may contribute to the observed association of increased long-term mortality and PM implantation [15]. 

Several studies have shown that balloon-expandable valves are associated with fewer conduction disturbances and lower PM rates [4, 16]. In our meta-analysis, balloon-expandable valves predominated, driven by the FRANCE-TAVI, SwissTAVI, and PARTNER 2 S3 cohorts. This may partly explain the overall low heterogeneity across studies and indicates that the prognostic impact of PM persists even in contemporary practice with improved device designs. However, the continued excess risk highlights that procedural refinements alone may not fully mitigate pacing-related harm.

The clinical implications are substantial. As TAVI expands to younger and lower-risk patients, long-term outcomes are increasingly important. The recently updated ESC/EACTS guidelines on the management of valvular heart disease now recommend TAVI as the preferred approach for patients aged ≥ 70 years, while surgical AVR remains the standard in younger patients [9]. With this age threshold, a growing number of patients with longer life expectancy will undergo TAVI, making the prevention of unnecessary PM implantation even more critical. Strategies to minimize conduction injury through careful valve positioning, device choice, and conduction-sparing techniques should be pursued [17]. Furthermore, novel pacing modalities such as His-bundle or left bundle branch pacing may help mitigate the adverse effects of conventional right ventricular pacing [18]. 

This study has limitations. All included studies were observational, with potential residual confounding. No causal relationship between PM implantation and long-term mortality can be established. Although most cohorts applied multivariable adjustment, covariate capture differed across registries, and unmeasured or incompletely measured factors may have contributed to residual confounding. Individual patient-level data were not available, precluding time-to-event analyses across cohorts.Variations in valve type, generation, and procedural technique may influence the observed effect. Follow-up completeness and endpoint definitions varied across registries, which may have affected comparability. To minimize bias from differential follow-up, we consistently used the explicitly reported 5-year mortality cutoff if reported. Finally, newer-generation valves introduced after 2022 were not yet represented, possibly underestimating the benefit of technological progress. The meta-regression is limited by the small number of studies, making the analysis underpowered and the null findings potentially unstable; nonetheless, the direction and magnitude of effects were consistent across all cohorts, supporting the overall robustness of the pooled estimate. Furthermore, Publication bias cannot be fully excluded, as funnel-plot inspection is insensitive with very few studies and formal tests were not feasible; however, the plot showed no visual asymmetry, suggesting no major distortion of the pooled effect.

## Conclusion

In summary, this meta-analysis demonstrates that permanent PM implantation after TAVI is associated with higher long-term mortality, even in the context of modern valve technology and improved procedural techniques. The findings underscore the importance of conduction-sparing approaches and physiologic pacing to preserve long-term survival after TAVI, particularly in the expanding population of younger, lower-risk patients.

## Supplementary Information

Below is the link to the electronic supplementary material.

Supplementary Material 1
