# Supplementary material for: Long-term outcome of patients undergoing pacemaker implantation after transcatheter aortic valve implantation: a systematic review and meta-analysis
Source: Cardiovasc Interv Ther. 2026 Jan 9;41(2):425–33. doi: 10.1007/s12928-025-01232-4 (PMC13002639; doi:10.1007/s12928-025-01232-4)
Supplement: Supplementary file 1 — Supplementary Material 1 [file 12928_2025_1232_MOESM1_ESM.docx]

Long-term outcome of patients undergoing pacemaker implantation after transcatheter aortic valve implantation: a systematic review and meta-analysis

Running Title: Pacemaker and Long-Term Mortality After TAVI

Cecilia Veraar^1,2^, Gudrun Lamm^1,2^, Lion Merl^1,2^, Arabella Fischer-Hammerschmied^3^, Matthias Granner^1,2^, Maximilian Will^1,2^, Konstantin Schwarz^1,2^, Andreas Kammerlander^4^, Julia Mascherbauer,^1,2^

**Affiliations:**

^1^Karl Landsteiner University of Health Sciences, Dr. Karl-Dorrek-Straße 30, 3500, Krems, Austria

^2^ Division of Internal Medicine 3, University Hospital St. Pölten, Dunant-Platz 1, 3100, St. Pölten, Austria

^3^Department of Anesthesiology, Intensive Care Medicine and Pain Medicine, Division of Cardiac Thoracic Vascular Anesthesia and Intensive Care Medicine, Medical University of Vienna, Vienna, Austria

^4^Department of Cardiology, Medical University of Vienna, Vienna, Austria

*The authors declare no conflicts of interest.*

**Correspondence to:**

Cecilia Veraar, MD

Karl Landsteiner University of Health Sciences, Krems, Austria

Department of Cardiology, University Hospital Sankt Poelten, Sankt Poelten, Austria

Dunant-Platz 1, 3100 St. Pölten

[cecilia.veraar@stpoelten.lknoe.at](mailto:cecilia.veraar@stpoelten.lknoe.at)

**Supplementary Table S1: Full electronic search strategy through August 30th, 2025**

| **PubMed** |
| --- |
| ("pacemaker"[Title]) AND ("transcatheter aortic valve implantation"[Title/Abstract] OR "TAVI"[Title/Abstract] OR "TAVR"[Title/Abstract]) AND ("mortality"[Title/Abstract] OR "survival"[Title/Abstract] OR "outcome"[Title/Abstract]) AND ("5 year"[Title/Abstract] OR "5-year"[Title/Abstract] OR "long-term"[Title/Abstract] OR "follow- up"[Title/Abstract]) |
| **EMBASE** |
| ('transcatheter aortic valve replacement'/exp OR 'transcatheter aortic valve implantation'/exp OR tavr OR tavi) AND (('survival analysis'/exp OR survival) AND rate OR mortality OR 'long-term survival' OR 'time-to-event') AND ((5 AND year* OR 'five year*' OR 10) AND year* OR 'ten year*' OR 'long term*' OR 'long-term follow-up') AND (pacemaker*:ti OR 'permanent pacemaker':ti OR 'pacemaker implantation':ti) AND (multicenter OR registry OR nationwide OR 'large cohort' OR 'consecutive patients' OR 'more than 300' OR '>300') AND [humans]/lim AND [abstracts]/lim AND ('article'/it OR 'article in press'/it) |

**Supplementary Table S2: Assessment of study quality using the Newcastle–Ottawa Scale (NOS)** Higher scores indicate lower risk of bias (maximum 9 points).

| Author (Year) | Representativeness of exposed cohort | Selection of non-exposed cohort | Ascertainment of exposure | Absence of outcome at baseline | Comparability of cohorts | Assessment of outcome | Follow-up long enough | Adequacy of follow-up | **Total** |
| --- | --- | --- | --- | --- | --- | --- | --- | --- | --- |
| Auffret (2024) FRANCE-TAVI | 1 | 1 | 1 | 1 | 2 | 1 | 1 | 1 | **9** |
| Chen (2024) PARTNER 2 S3 | 1 | 1 | 1 | 1 | 1 | 1 | 1 | 0 | **7** |
| Rück (2021) SWEDEHEART | 1 | 1 | 1 | 1 | 2 | 1 | 1 | 0 | **8** |
| Badertscher (2025) SwissTAVI | 1 | 1 | 1 | 1 | 2 | 1 | 1 | 1 | **9** |
| Wasim (2025)  TAVI-NOR | 1 | 1 | 1 | 1 | 1 | 1 | 1 | 0 | **7** |
| Hochstadt (2021)  Tel-Aviv | 1 | 1 | 1 | 1 | 1 | 1 | 1 | 0 | **7** |
| Myat (2021)  UK-TAVI | 1 | 1 | 1 | 1 | 2 | 1 | 1 | 0 | **8** |
